# Pattern of Participation in Colorectal Cancer Screening from a Population-Based Screening Program in Iran

**DOI:** 10.34172/aim.31072

**Published:** 2024-08-01

**Authors:** Farimah Rahimi, Reza Rezayatmand, Elaheh Najafi, Zahra Ravankhah, Elham Tabesh, Peyman Adibi

**Affiliations:** ^1^Health Management and Economics Research Center, Isfahan University of Medical Sciences, Isfahan, Iran; ^2^School of Public Health, Tehran University of Medical Science, Tehran, Iran; ^3^Cancer Registry of Health Deputy, Isfahan University of Medical Sciences, Isfahan, Iran; ^4^Isfahan Gastroenterology and Hepatology Research Center (IGHRC), Isfahan University of Medical Sciences, Isfahan, Iran

**Keywords:** Colorectal cancer screening, Fecal immunochemical test, Iran, Isfahan, Participation rate

## Abstract

**Background:**

In Isfahan, the fecal immunochemical test (FIT) has been used since January 2016 as part of the Iran’s Package of Essential Non-communicable Diseases (IraPEN) program for colorectal cancer (CRC) screening. The test is recommended for people who are 50-70 years old. Then, those with positive results would be referred for colonoscopy. This study aims to describe the uptake of the program and its outcome.

**Methods:**

A retrospective observational study was performed by collecting data from Isfahan Vice-Chancellor for Health database for this study purpose. The number of participators, the number of positive FIT, and the number of detected polyps or cancers were determined.

**Results:**

Between 2016 and 2019, the number of participants in the program reached 345 207 individuals (nearly 40% of the eligible population of 874 674). Totally, 21 264 participants (6.1%) had positive tests, of whom about 20% underwent the recommended colonoscopy with available reports, and 971 (24%) and 110 (3%) patients were diagnosed with polyps and CRC, respectively.

**Conclusion:**

Over four years of screening with FIT in Isfahan, 40% of the eligible population participated. Among those with positive FIT results, 20% underwent colonoscopy, and approximately 26% of these individuals were identified as having polyps or cancer. This study provides valuable insights into the uptake and outcomes of a population-based CRC screening program in Isfahan, Iran. The findings highlight the need for targeted interventions to increase participation rates and improve the detection of polyps and CRC cases.

## Introduction

**Figure 1:**
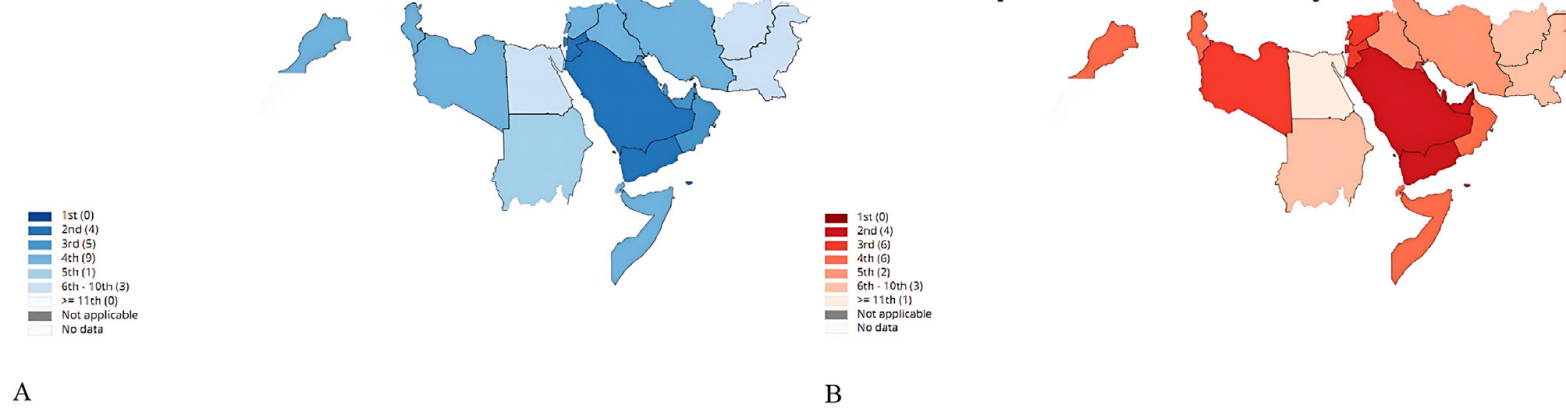
Colorectal cancer in the WHO Eastern Mediterranean Region in 2020. (A) Estimated age-standardized incidence rates in 2020, colorectum, both sexes, all ages. (B) Estimated age-standardized mortality rates in 2020, colorectum, both sexes, all ages. Source: Global Cancer Observatory 2020, International Agency for Research on Cancer, World Health Organization.^3^

 Colorectal cancer (CRC) is the third leading cause of cancer-related deaths worldwide, resulting in approximately 935,173 deaths in 2020.^1^ According to the World Health Organization (WHO), in 2020, the incidence, prevalence, and mortality rates of CRC in the world were 19.5, 67.4, and 9 in 100 000, respectively.^2^ In the Eastern Mediterranean Region, CRC is the third and fifth most common cancer in terms of incidence and mortality, with 50 403 new cases and 27 975 deaths in 2020 (Figure 1).^3^ Reports from Iran show an increasing trend of CRC^4^ which is becoming one of the most common cancers of the gastrointestinal tract and the third most common cancer with an estimated 6220 deaths in 2020.^5^

 With advancing age, the risk of developing CRC increases, such that more than 90% of CRC cases occur in persons aged 50 or older.^6^ Also, those over 65 years of age are about 30 times more likely to be diagnosed with CRC than those aged 25–49 years in the US.^7^ Similarly, the incidence and prevalence among Iranians aged 50-74 years are estimated at 69.3 and 184 in 100 000, respectively and the mortality rate is 28.6 in this age group.^5^

 Studies show that early detection interventions can reduce CRC-related deaths by about 15–33%.^8^ Thus, there is a need for preventive policies against this disease. CRC screening aims to reduce mortality by detection of colonic adenomas and early CRCs.

 In 2015, screening for CRC was launched in Iran. It was based on a national screening program that also covered screening for breast and cervical cancers. CRC screening was first piloted in four provinces of the country including Isfahan. Since the implementation of the CRC screening program in January 2016, fecal immunochemical test (FIT) has been requested for individuals aged 50–70 years who come to the health centers. If the FIT is positive, participants are encouraged to undergo a colonoscopy in hospitals or public health centers/private clinics.

 For a successful and beneficial screening method, high participation rates are necessary and the population acceptability of the test affects compliance.^9^ So, in this study, we try to analyze the participation rate of the CRC screening program in Isfahan, Iran, to determine its success and usefulness.

## Materials and Methods

 We performed a retrospective observational study. Individuals aged 50-70 years who had been registered on the E-integrated health information system (SIB (in Persian)) database and had visited the public health centers in the Isfahan province at least once, were included in the study. Rate of adherence to the CRC screening program was assessed.

###  Isfahan CRC Screening Program

 The CRC screening program in Isfahan is a part of the national IraPEN (Iran’s Package of Essential Non-communicable Diseases) program which was first launched in 2016 as a pilot program and has been continuing since then.

 In this program, all individuals aged 50–70 years in Isfahan were screened for early diagnosis of CRC. For this purpose, the FIT test was recommended to the target the population with no history of CRC in the first- and second-degree family members and no history of polyps, inflammatory bowel disease (IBD), and bleeding. There are no personal invitations for participation in the CRC screening program and anyone in the target group who has been referred to the health centers is recommended to participate in the CRC screening program. Initial training is given to the person to perform the test and deliver it at his/her next visit. Participants whose sample is not assessable (outdated test, error in filling out the form, and so on) are sent a new test. After analysis, if the result of the test is negative, he/she is told to repeat the test two years later. However, those with a positive test are referred for colonoscopy in public or private hospitals. The results of referred colonoscopies or pathology reports (if applicable) are sent back to the health centers and recorded on each individual’s profiles.

###  Data Collection

 All 50 to 75-year-old people who lived in Isfahan between 2016 to 2019 and were registered on the SIB system were eligible to take part in the CRC screening program. Data were extracted from SIB databases managed by the Vice-Chancellor for Health, Isfahan University of Medical Sciences. Required data were obtained from four datasheets as follows:

1. Information of all people born from 1946 to 1969, who are registered in the SIB system of Isfahan,
2. Information of all individuals who performed a FIT test between 01/01/2016 and 30/12/2019 in Isfahan,
3. Result of the analysis of the FIT tests
4. Information pertaining to follow-up on the positive FIT tests and the results of their colonoscopy.

Variables included in this study were participation in the CRC screening program, the results of FIT and colonoscopy, variables related to the socioeconomic status of the participants (i.e. age, gender, education level, and job), and variables related to the risk factors and health status of the participants.

###  Analysis

 Four series of data mentioned earlier were merged using Excel and STATA software and the data were analyzed to report the descriptive statistics. Also, all tests were performed according to the relevant guidelines and regulations of the research ethics committee of Isfahan University of Medical Sciences.

## Results

 The target population was about 874 674 individuals aged 50–70 years during the implementation of the CRC cancer screening program (2016-2019) and registered in the SIB system. The mean age of the target population was 59.97 ± 6.22 and the male-to-female ratio was 1.02. About 40% of this population took part in the CRC screening program. The mean age of participants was 59.91 ± 5.38 and most of them were women (about 56%). There were more female participants while non-participants were more frequently male. The proportion of individuals with elementary education or less was higher among participants than non-participants (76.60% vs. 61.57%). At the same time, the proportion of individuals with higher education (high school and more) was higher among non-participants than participants (33.76% vs. 19.49%). Regarding employment status, the unemployed were more common among non-participants. Individuals without insurance were more frequent among non-participants (5.81% vs. 3.81%). We categorized the weight based on body mass index (BMI), and found that the proportion of overweight or obese people was much higher among participants than non-participants (57.80 % vs. 16.37%). Participants in the CRC screening program had on overage more children than non-participants. More details are shown in Table 1.

**Table 1:** Descriptive Statistics of Participants and Non-participants in the CRC ScreeningProgram.

**Variable**		**Participants**	**Non-participants**
		**Frequency (Percent)**	**Frequency (Percent)**
Gender	Female	195160 (56.53)	236281 (44.63)
	Male	150047 (43.47)	293186 (55.37)
Education	Elementary and less	264439 (76.60)	325975 (61.57)
	High school	47394 (13.73)	117816 (22.25)
	College/undergraduate/graduate	19884 (5.76)	60945 (11.51)
	Unknown	13490 (3.91)	24731 (4.67)
Job	Employed	317961 (92.11)	434872 (82.13)
	Unemployed	19480 (5.64)	55105 (10.41)
	Missed	7766 (2.25)	39490 (7.46)
Living status	Alone	37920 (10.98)	65183 (12.40)
	With partner	304857 (88.31)	463796 (87.60)
	Missed	2430 (0.71)	24 (0)
Health insurance	Yes	329293 (95.39)	491787 (92.88)
	No	13167 (3.81)	30775 (5.81)
	Missed	2747 (0.80)	6905 (1.30)
Weight	Normal	145675 (42.20)	442786 (83.63)
	Overweight	115489 (33.45)	50695 (9.57)
	Obesity	84043 (24.35)	35986 (6.80)
Tobacco products record	No	309722 (89.72)	142992 (27.01)
	Yes	10926 (3.17)	4437 (0.84)
	Missed	24559 (7.11)	382038 (72.16)
		**Mean (Std. Dev.)**	**Mean (Std. Dev.)**
Age	Year	59.91 (5.38)	60.90(7.35)
Number of children		3.33 (1.91)	2.57(1.69)

 There were 21 264 positive and 269 178 negative tests, yielding a positive rate of 6.1%. The rest of the tests were non-assessable and the rate of non-assessable tests was 16%. As depicted in Table 2, in the positive FIT group, the proportion of men was approximately equal to women (1.03: 1), but in the non-assessable group, women held a larger share (with a man/woman ratio of 0.66:1). More details are presented in Table 2.

**Table 2:** Descriptive Statistics of FIT Test in the CRC ScreeningProgram

**Variable**		**Positive test**	**Negative test**	**Non-assessable test**
		**Frequency (Percent)**	**Frequency (Percent)**	**Frequency (Percent)**
Gender	Female	10800 (50.79)	151324 (56.22)	33036 (60.32)
	Male	10464 (49.21)	117854 (43.78)	21729 (39.68)
Education	Elementary and less	15781 (74.21)	206751 (76.81)	41907 (76.52)
	High school	2813 (13.23)	37790 (14.04)	6791 (12.40)
	College/undergraduate/graduate	1115 (5.24)	15849 (5.89)	2920 (5.33)
	Unknown	1555 (7.32)	8788 (3.26)	3147 (5.75)
Job	Employed	19169 (90.15)	248375 (92.27)	50417 (92.06)
	Unemployed	1384 (6.51)	14988 (5.57)	3108 (5.68)
	Missed	711 (3.34)	5815 (2.16)	1240 (2.26)
Living status	Alone	2301 (10.76)	239385 (88.93)	47641 (86.99)
	With partner	17831 (83.86)	29279 (10.87)	6549 (11.96)
	Missed	1132 (5.32)	514 (0)	575 (1.05)
Health insurance	Yes	19722 (92.75)	257754 (95.76)	51817 (94.62)
	No	689 (3.24)	10011 (3.72)	2467 (4.50)
	Missed	853 (4.01)	1413 (0.52)	481 (0.88)
Weight	Normal	8746 (41.13)	97251 (39.84)	29678 (54.19)
	Overweight	6894 (32.42)	94235 (35.01)	14360 (26.22)
	Obesity	5624 (26.45)	67692 (25.15)	10727 (19.59)
Smoking	No	18079 (85.02)	241749 (89.81)	49894 (91.11)
	Yes	801 (3.77)	9200 (3.42)	925 (1.69)
	Missed	2384 (11.21)	18229(6.77)	3946 (7.21)
		**Mean (Std. Dev.)**	**Mean (Std. Dev.)**	**Mean (Std. Dev.)**
Age	Year	60.74 (5.37)	59.68 (5.41)	60.71 (5.09)
Number of children		3.38 (1.93)	3.32 (1.90)	3.37 (1.94)

 In the positive test population, only about 19% performed the required colonoscopy with available reports. The main reasons mentioned for not performing a colonoscopy were: fear and unwillingness (43%), financial and access problems (43%), lack of physicians’ recommendations (8%), and migration (6%). As depicted in Table 3, the proportion of men among those who underwent colonoscopy was higher than the proportion of men among those who did not undergo colonoscopy. The share of individuals who did not live alone was higher in the colonoscopy group than in the non-colonoscopy group.

**Table 3:** Descriptive Statistics of Following the Positive FIT Tests in the CRC Screening Program

**Variable**		**Colonoscopy**	**No colonoscopy **
		**Frequency (Percent)**	**Frequency (Percent)**
Gender	Female	1888 (46.79)	3271 (50.24)
	Male	2147 (53.21)	3240 (49.76)
Education	Elementary and less	2278 (56.34)	5350 (82.17)
	High school	522 (12.91)	707 (10.86)
	College/undergraduate/graduate	224 (5.54)	272 (4.18)
	Unknown	1011 (25.01)	182 (2.80)
Job	Employed	3531 (87.49)	6317 (97.02)
	Unemployed	259 (6.40)	190 (2.92)
	Missed	245 (6.11)	4 (0.06)
Living status	Alone	384 (9.50)	5662 (86.96)
	With partner	2724 (67.38)	751 (11.53)
	Missed	927 (23.12)	98 (1.50)
Health insurance	Yes	3194 (79.00)	6275 (96.38)
	No	76 (1.88)	211 (3.24)
	Missed	765 (19.12)	25 (0.38)
Weight	Normal	2063 (51.23)	2648 (40.67)
	Overweight	1078 (26.66)	2170 (33.33)
	Obesity	894 (22.11)	1693 (26.00)
smoking	No	2864 (70.98)	5891 (90.48)
	Yes	104 (2.57)	253 (3.89)
	Missed	1067 (26.44)	367 (5.64)
		**Mean (Std. Dev.)**	**Mean (Std. Dev.)**
Age	Year	61.03 (5.66)	60.75 (5.26)
Number of children		3.30 (1.92)	3.38 (2.01)

**Figure 2:**
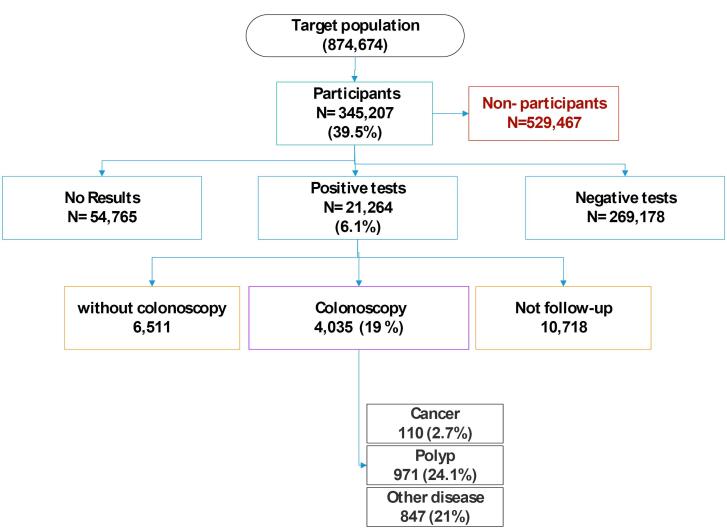
Study Participation Flow Diagram Over One Round of Screening

 The results of colonoscopy for the positive FIT group show that 971 (24.1%) and 110 (2.7%) individuals were found to have as polyp and CRC, respectively. All participation rates over one round of screening are shown in Figure 2.

## Discussion

 This study aimed to describe the implementation of the CRC national screening program in Isfahan. Our study showed a participation rate of about 40% in the CRC screening program in Isfahan. Other studies reported this rate at 54.4% in Italy, 60.4 % in Slovenia,19.9% in Croatia, and 22.7% in the Czech Republic.^10^ It should be noted that a formal personal invitation for screening was not usually made, and the FIT test was performed for individuals who randomly visited the health centers for other reasons. Thus, sending a formal personal invitation to the target group can involve a larger number of the population in the program.^8^

 In the present study, the participation rate of women was higher than men (approximately 57%). This finding is in line with other studies showing the tendency for greater participation among women.^11^ According to the results of the present study, married people have a higher participation rate than single people (about 88%). The results of other studies have also found similar results. For example, the study by Bocci et al found that unmarried people are less inclined to participate in CRC screening tests, concluding that the invitation of married people in pairs can facilitate CRC screening.^12^

 According to a study by Carrasco-Garrido et al in Spain and Wools et al, higher levels of education have increased the use of preventive services and CRC screening rates.^13,14^ In contrast, we found that people with low education levels participated more frequently in the screening program. This could be due to the greater availability of time for visiting the health center for those with low education compared to those with higher education.

 One significant limitation of the CRC screening project, in addition to the 40% participation rate, was the low rate of colonoscopy follow-up. Only about 19% of individuals with positive FIT results proceeded to undergo colonoscopy, which is substantially lower than reported in previous studies and international benchmarks. For instance, Anna Pellat et al reported that 70.5% of the participants with positive results had a colonoscopy afterward.^15^ Another study showed that about 95% of people undergo colonoscopy.^14^ The rate of participation in colonoscopy screening reported in four European countries (Norway, Sweden, Poland, and the Netherlands) ranged between 22.9% and 60.7%.^16^ A study in Italy with 26,703 subjects for one-time colonoscopy and 26 599 subjects with FIT every 2 years showed that the rate of participation in colonoscopy and FIT groups was 24.6% and 34.2%, respectively.^17^ In a study conducted in China in 2020, the participation rate for colonoscopy was reported to be 15.6%, which was lower than in the United States, Canada, and Australia.^18^ In the study by Tastan et al, 160 people with the condition were studied and 18 of these people (11.3%) refused to have a colonoscopy.^19^ In our study, the percentage of men who underwent colonoscopy was higher (53%). However, in other studies, different results were obtained. For example, a study by Kolligs et al found that women were more likely than men to have a colonoscopy.^20^ Regarding other characteristics, one study showed that people with lower education had less participation in colonoscopy screening,^21^ which is not in line with the results of the present study. In terms of risk factors, our study showed that smokers underwent colonoscopy less frequently than non-smokers. However, in a study by Zhang et al, different results were reported, with people with a history of smoking, a low-fiber diet, high red meat intake, and low physical activity being more likely to participate in screening.^22^ Further studies are needed to confirm these results.

 The colonoscopy rate in Isfahan is similar to other Iranian CRC screening project studies and is unacceptably low. This issue is exacerbated by comparing our findings with higher follow-up rates reported in various studies and countries. To improve the effectiveness of CRC screening, addressing the barriers to follow-up colonoscopy is crucial, which may include factors such as patient education, access to healthcare services, logistical challenges, and fears or misconceptions about the procedure.

## Conclusion

 CRC is the third most common cancer worldwide. Therefore, early detection of this disease reduces its mortality and financial burden. In Isfahan, the FIT has been used since January 2016 as a part of Iran’s Package of Essential Non-communicable Diseases (IraPEN) program for CRC screening. The test is recommended for people who are 50-70 years old. Then, those with positive results would be referred for colonoscopy. In the current study, we described results regarding the implementation of the program. Totally, 40% of the target population participated in the program. Among these, 19 % had a positive FIT, of whom about 19% underwent colonoscopy. In total, we found 110 cases of CRC (2.7%) and 971 cases of polyps (24%). Women, married individuals, those with lower education, and employees participated more frequently in the screening with FIT. The findings of our study would help health professionals to increase the uptake of the program which results in early detection of more CRC cases in the future.
